# Bipolar Radiofrequency-Assisted Liposuction for Cervical Contouring in Eastern Asians

**DOI:** 10.1093/asjof/ojad035

**Published:** 2023-04-04

**Authors:** ShihChun Yen, JiGeng Wang, Xiang Gao, QiuXuan Zhu, CaiYing Song, Fei Zhu

## Abstract

**Background:**

East Asian beauty standards uphold a V-shaped face and a long slender neck. Some patients are dissatisfied with the concurrent nonsurgical treatment and prefer limited downtime with minimally invasive procedures to achieve a natural skin-tightening outcome. The authors performed bipolar radiofrequency-assisted liposuction (RFAL) to achieve cervical rejuvenation.

**Objectives:**

To evaluate the efficacy and safety of RFAL for the treatment of cervical skin and soft-tissue laxity in Eastern Asians.

**Methods:**

In total, 66 patients with neck skin and soft-tissue laxity were treated with bipolar RFAL under tumescent local anesthesia. Further, the surgical outcomes were evaluated based on patient satisfaction score and the Global Aesthetic Improvement Scale (GAIS) score at 6 months postoperatively. Moreover, the incidence of postoperative complications was determined.

**Results:**

All patients were followed up for at least 6 months. After RFAL technologies treatment, significant improvement in the neck contour was observed. The average GAIS score was 3.03 (4, very much improved; 3, much improved; 2, improved; 1, no change; and 0, worsened). Approximately 93% of patients were satisfied with the RFAL neck contouring outcome. Notably, no serious complications requiring further intervention were encountered in this series.

**Conclusions:**

The described RFAL treatment significantly improved the refinement of neck contouring in Eastern Asian subjects. The simple, minimally invasive cervical procedure under local anesthesia improve the cervical-mental angle definition, tissue-tightening effect, face slimming, and the mandibular line. No serious adverse events except mild complications were reported. This treatment could achieve extraordinary results with a high safety profile.

**Level of Evidence: 4:**

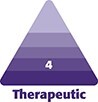

Owing to the use of video technology during the COVID-19 pandemic, which increased the time spent seeing one's face on screen, increasing number of people became fixated on perceived physical imperfections and noticed facial profiles that they would like to change. The beauty standards in Eastern Asia uphold perfection, which is often characterized by a V-shaped face and a long slender neck. The ideal neck configuration is often described as having a submental-cervical angle of 90° to 105°, the definition and sharpness of the mandibular angle, the prominence, a well-defined inferior and posterior gonial angle trough, and a distinct mandibular inferior border.^1,2^ With age, there is an increase in skin and connective-tissue laxity and fat accumulation in the submental and submandibular regions, making the face look flabby or droopy; moreover, the cervicomental angle becomes obtuse. Changes in the neck due to aging can alter the natural youthful facial curvatures and side profiles. This has led to patients in East Asia undergoing aesthetic procedures to achieve a sharp lower face shape and the geometric angle between the cylindrical neck and the straight-line inferior border of the jaw. There are different treatment options for managing the abovementioned issues. These include facelifting, cervicoplasty, thread lifting, submental liposuction, deoxycholic acid injection, ultrasound energy, cryotherapy, neuromodulator injection, microneedling, radiofrequency (RF) energy skin tightening, and laser skin resurfacing.

A full facelift with cervicoplasty is considered the gold standard for patients with severe neck and lower face skin laxity. However, there is an increase in the number of young patients in their late twenties or thirties who require aesthetic improvement. The skin and soft-tissue laxity of people in this age group is often not severe enough to justify traditional excision procedures; in addition, increasing the number of patients prefers limited downtime with minimally invasive procedures to achieve a natural result. Additionally, many patients have become dissatisfied with concurrent nonsurgical treatments and instead require a more significant aesthetic improvement.

In general, neck rejuvenation primarily involves tightening of the skin and soft tissue and removal of an appropriate amount of fat deposits. Submental liposuction is an effective and minimally invasive method that can significantly improve the neck contour of some patients who have submental localized fat and adequate skin elasticity and muscle tone.^3-5^ However, due to the acuteness of the cervicomental angle, liposuction alone can have poor aesthetic outcomes if the support of the overlying skin is inadequate particularly in individuals aged over 40 years and those with recessed chins and microgenia.

In 2009, bipolar RF-assisted liposuction (RFAL; BodyTite system, InMode, Irvine, CA) was first adopted for body contouring in clinical practice.^6^ Moreover, the new handpieces (NeckTite, FaceTite, Accutite; InMode, Irvine, CA) were designed for fine body parts based on the Bodytite system. Emerging RFAL technologies take advantage of a tissue's inherent characteristics to facilitate liposuction and achieve concomitant tissue tightening. Bipolar RFAL applies RF energy to target the upper dermal collagen network, deeper fascia layer, and fibrofatty septum. As the local temperature within the dermis, subcutaneous tissue, and fibrofatty septum reaches 65°C, collagen is denatured, leading to immediate collagen contraction and the stimulation of neocollagenesis.^7-9^ These thermal effects optimize skin and soft-tissue contraction to improve aesthetic outcomes, and face and neck rejuvenation has been attracting more attention in recent years.^7,10,11^

However, a recent systematic review of subsurface RF treatments conducted by Swanson demonstrated that the patient safety profile and efficacy of RFAL treatment remained doubtful; thus, further studies are required to verify these parameters.^12^ This study aims to assess the effectiveness and safety of RFAL applied for neck contouring in Eastern Asians.

## METHODS

### Subjects

**Figure 1.**
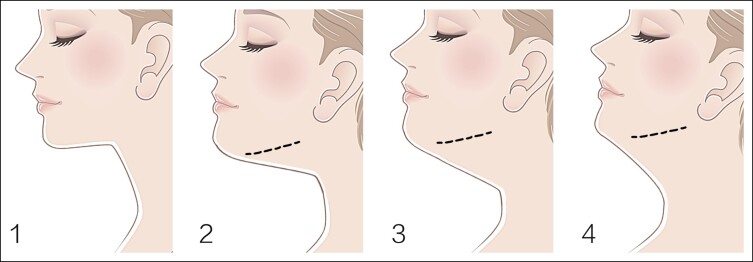
Knize's classification of cosmetic deformity of the neck. Grade I: normal cervicomental angle; Grade II: mild; Grade III: moderate; Grade IV: severely oblique cervicomental angle.

This retrospective case series was performed from June 2021 to April 2022. In total, 66 Eastern Asian patients (60 females and 6 males, aged 23 to 62 [mean: 34] years) with neck skin and soft-tissue laxity and Knize II to IV neck contour who wanted neck rejuvenation were included in this study. According to the classification proposed by Knize (Figure 1), 60 patients were classified as Grades II and III, mild-to-moderate cervicomental angle deformity; 4 patients as Grade III, moderate cervicomental angle deformity; 2 patients as Grade IV, a severely oblique cervicomental angle.^13^

All patients were treated with RFAL using bipolar RF energy devices (InMode bipolar RF system, FaceTite), and the procedures were conducted by 2 experienced plastic surgeons (SC.Y. and JG.W.). All study participants provided their written informed consent. This study was conducted in accordance with the principles of Declaration of Helsinki, and all participants gave their written informed consent for the use and analysis of their data.

Patients who sought aesthetic neck contours for mild-to-moderate skin and soft-tissue laxity of the cervicomental area were included in this study. Our exclusion criteria were as follows: (1) chronic illness; (2) pregnancy or breastfeeding; (3) the presence of an installed pacemaker or internal defibrillator; (4) a history of surgery within 3 months; (5) the presence of severe platysma bands; and (6) nonprovision of one's informed consent to participate in the study or inability to complete postoperative follow-up. Clinical follow-up was conducted on Postoperative Days 1, 7, 30, 90, and 180. Images were taken in frontal, lateral, and oblique views. To evaluate procedural outcomes, the results were evaluated using the Global Aesthetic Improvement Scale (GAIS; 4, very much improved; 3, much improved; 2, improved; 1, no change; and 0, worsened), which was used as a reference tool (Table 1). The value recorded was determined independently by 2 plastic surgeons (X.G. and QX.Z.). The patient's subjective satisfaction was assessed using a 4-point scale (1, poor; 2, fair; 3, good; and 4, excellent). The complications that required medical intervention, such as hematomas, nerve damage, and seromas, or that were transient in nature, such as short-duration neuropraxia, subcutaneous induration, and burns, were documented.

**Table 1.** The Global Aesthetic Improvement Scale

Score	State	Description
4	Very much improved	The ideal result has been achieved
3	Much improved	The result is markedly improved but not optimal
2	Improved	The result is improved but an additional procedure is recommended
1	No change	The result is the same compared with the preoperative state
0	Worse	The result is worse compared with the preoperative state

### Anesthesia

All 66 patients underwent bipolar RFAL under tumescent local anesthesia. No sedation or general anesthesia was used.

### Preoperative Markings

Preoperative marking was performed with the patient in the standing position. Preoperative markings were pentagon shaped, extending from the whole submandibular region to the neck area along with the sternocleidomastoid muscle (SCM) anterior border down to the level of the thyroid cartilage. Submental and submandibular fat deposits were marked, and landmarks of the mandibular border, SCM, and gonial angle were identified. Pinch tests were used to draw an exact topographic map. Further, asymmetry in fat distribution was carefully noted using the pinch test.

### Surgical Technique

The patient was placed in the supine position with the head hyperextended. Then, 800 mg of lidocaine and 1 mg of epinephrine in 1 L of normal saline constituted the tumescent solution. Puncture incisions were made using a 16-G needle in the submental crease directly below the chin and 2 cm below the earlobes in the lateral neck at the anterior border of the SCM for lateral liposuction. Then a 25-G spinal needle was used to slowly infiltrate the tumescent solution into the subcutaneous plane. Careful and gentle tumescent infiltration elevated the subcutaneous fat away from the deeper neurovascular structures below the platysma muscle. This maneuver minimized the risk of injury to the marginal mandibular nerve. Surgery started 15 min after tumescent infiltration when the adrenaline started having an effect and the skin became pale. Liposuction (SAL) was initially performed using a facial semi-blunt microcannula set (HK Surgical, San Clemente, CA). SAL is performed under constant visual and manual control, and submandibular and submental fat pads must be fully addressed. After 5 to 10 min of liposuction, a sterile ultrasound gel was used to cover all the treated areas. We used a bipolar RF device (InMode bipolar RF system, FaceTite) with settings at a target temperature of 38°C to 40°C cutoff for the external probe and a 60°C to 70°C cutoff for the internal probe.

The FaceTite handpiece, which can be deployed in a repeated retrograde-slide movement at the tunnels created by liposuction, stopped within 1 cm of the access port to prevent overheating of the area. The RF handpiece used a fan pattern of heating movement from the access point and systematically heated the predetermined treatment areas. Caution must be taken to maintain the inner probe superficial to the platysma muscle to prevent nerve and gland injury. The endpoint was reached when subdermal and epidermal surface temperatures were obtained. The common target temperatures of the neck were 38°C externally and 65°C internally. The treatment time of FaceTite was ∼5 to 10 min in the central neck area and per lateral neck area on average. Finally, SAL was performed again for contour refinement and the removal of residual fluid and liquefied fat and tumescent fluid. All patients underwent open drainage to minimize postoperative bruising and edema, after which they were instructed to wear moderate-compression garments for 72 h continuously. The patients were followed regularly on Postoperative Days 1, 7, 30, 90, and 180.

### Collection Data and Outcome Evaluation

Participants’ demographic data and clinical characteristics were obtained from the electronic medical records. The collected variables included age, sex, BMI, liposuction volume (total aspirate volume), RF application energy, the bipolar RF device's external temperature and internal temperature settings, the tumescent fluid injected, and the duration of surgery. The postoperative outcomes were investigated using the GAIS score, which was used as a reference parameter (Table 1). The value recorded was determined independently by 2 independent plastic surgeons (X.G. and QX.Z.). All patients were instructed to rate cervical region improvement using a 4-point scale (1, poor; 2, fair; 3, good; and 4, excellent) on Day 180 of the visit. The patient's subjective effectiveness rate was calculated as ([excellent + good]/total sample size) × 100%. In addition, complications such as hematoma, infection, nerve damage, and seroma or those that were transient in nature, such as neuropraxia of short duration, subcutaneous induration, and burns were recorded.

## RESULTS

### Clinical Characteristics and Treatment Effects

Participants’ clinical characteristics and treatment outcomes are shown in Table 2. The described technique was used for 66 patients with a mean age of 35.64 (range, 23-62) years; 60 patients (90.91%) were females and 6 patients (9.09%) were males. The authors recorded the total aspiration volume instead of the fat volume because a volume of no more than 10 to 15 mL (based on clinical findings at the time of liposuction of the supraplatysmal fat) is challenging to quantify.^14^ Further, the post-RF application area contains lots of mixtures of liquefied fat and tumescent fluid, making it more difficult to quantify.

**Table 2.** Summary Table of Clinical Characteristics of Enrolled Patients Treated With Radiofrequency-Assisted Liposuction

Variable		Number	Median	Minimum	Maximum	Mean	Standard deviation	Percent
Age		66	35.0	23.0	62.0	35.64	8.00	NA
BMI		66	22.95	18.5	28.4	22.57	2.55	NA
Total energy (kJ)		66	6.25	5.0	12.0	6.41	1.10	NA
Operative time (min)		66	55.0	30.0	90.0	58.33	10.46	NA
Total volume of aspirate (mL)		66	25.0	10.0	60.0	26.06	12.41	NA
Volume of tumescent fluid injected (mL)		66	130.0	90.0	220.0	138.03	30.45	NA
External temperature (°C)		66	39.25	38.0	40.0	39.01	0.79	NA
Internal temperature (°C)		66	68.0	60.0	70.0	67.11	1.97	NA
Patient satisfaction score		66	3.5	1.0	4.0	3.41	0.69	NA
GAIS doctor 1		66	3.0	2.0	4.0	3.05	0.44	NA
GAIS doctor 2		66	3.0	2.0	4.0	3.02	0.53	NA
Sex	Male	6	NA	NA	NA	NA	NA	9.09
	Female	60	NA	NA	NA	NA	NA	90.91
Treatment area (%)	Lower mandible	60	NA	NA	NA	NA	NA	90.91
	Lower mandible and lower face	6	NA	NA	NA	NA	NA	9.09
Patient rating satisfaction (%)	Excellent	33	NA	NA	NA	NA	NA	50.00
	Good	29	NA	NA	NA	NA	NA	43.94
	Fair	2	NA	NA	NA	NA	NA	3.03
	Poor	2	NA	NA	NA	NA	NA	3.03
Complications (%)	None	53	NA	NA	NA	NA	NA	80.30
	I-II degree burns	5	NA	NA	NA	NA	NA	7.58
	Nodules during recovery	5	NA	NA	NA	NA	NA	7.58
	Neurapraxia	3	NA	NA	NA	NA	NA	4.55

Satisfaction score 4-point scale (1, poor; 2, fair; 3, good; and 4, excellent). GAIS score: 4, very much improved; 3, much improved; 2, improved; 1, no change; 0, worsened. GAIS, Global Aesthetic Improvement Scale; NA, not applicable.

The median BMI was 22.57. The treatment area was the lower mandible (submental/submandibular area) in 60 patients (90.91%) and the lower mandible and lower face area in the remaining 6 patients (9.09%). The mean amount of RF energy delivered was 6.41 (range: 5-12) kJ. The mean total aspiration volume was 26.06 mL, and the mean volume of tumescent fluid injected was 138.38 mL. The maximum external temperature of the RF device was 40°C, the internal temperature was 70°C, and the average duration of surgery was 58.33 min.

**Figure 2.**
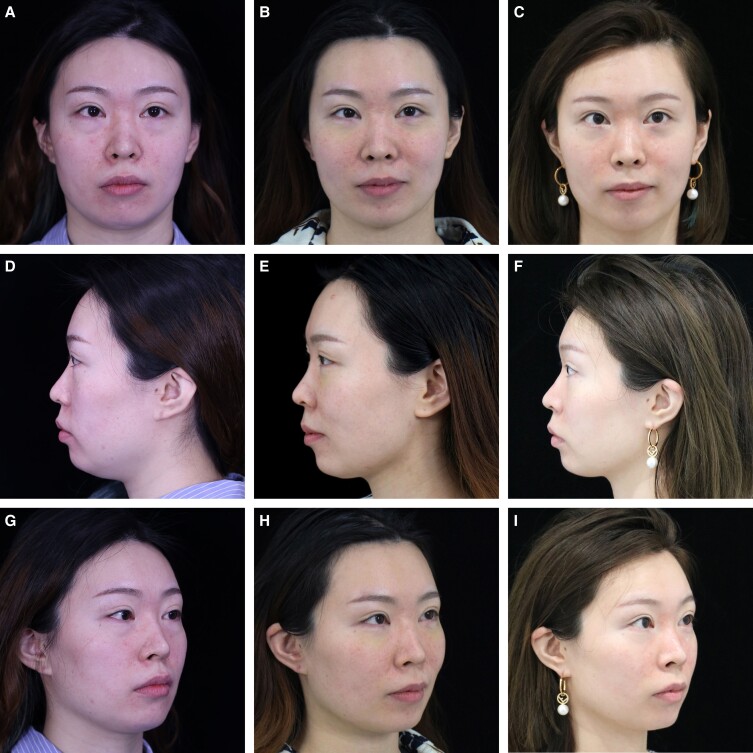
A 31-year-old female patient with blunting of the jawline and cervical-mental angle, microgenia, and skin ptosis shown at frontal, lateral, and oblique views at (A, D, G) preoperative, (B, E, H) 6 months after, and (C, F, I) 12 months after the neck radiofrequency-assisted liposuction procedure.

**Figure 3.**
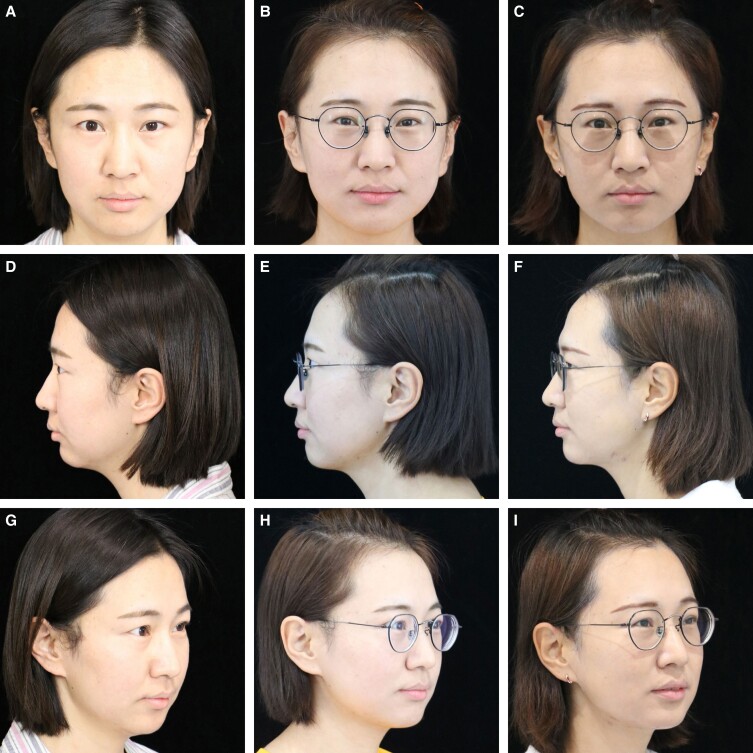
A 32-year-old female patient with blunting of the jawline and cervical-mental angle, jowling, and skin ptosis shown at frontal, lateral, and oblique views at (A, D, G) preoperative, (B, E, H) 6 months after, and (C, F, I) 12 months after the neck radiofrequency-assisted liposuction procedure.

**Figure 4.**
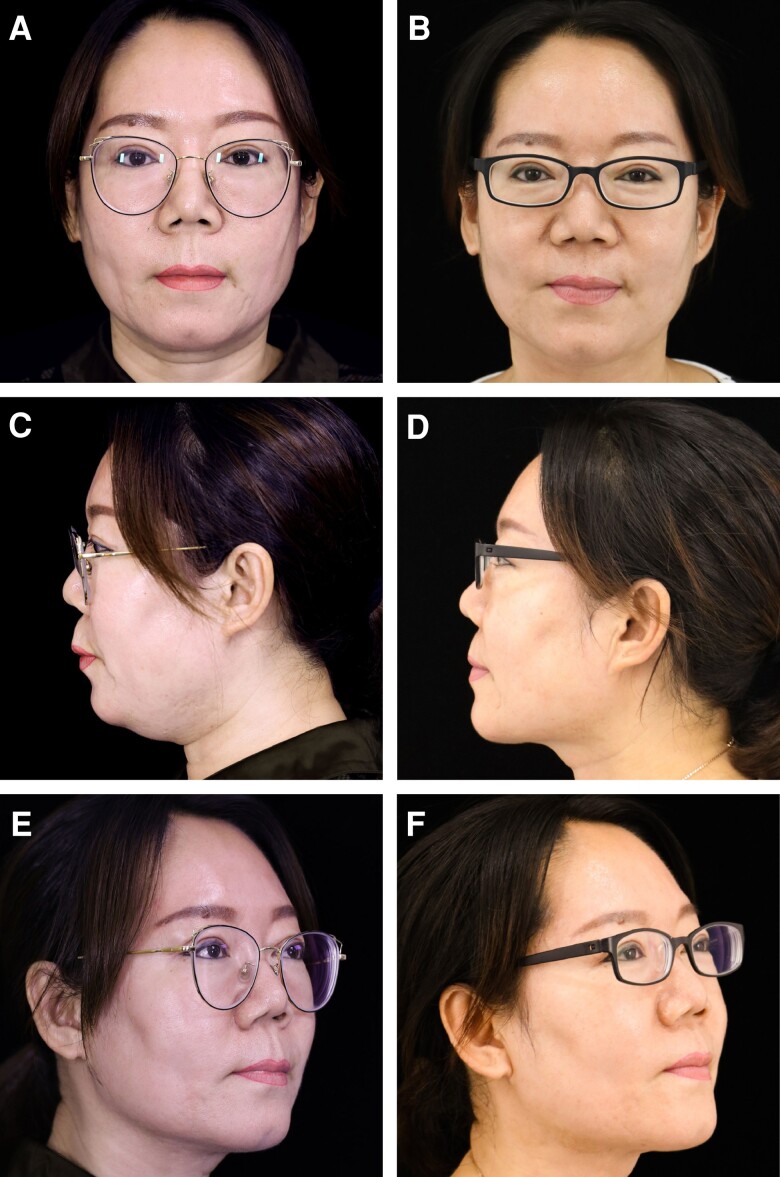
A 47-year-old female patient with blunting of the jawline and cervical-mental angle and moderate skin ptosis shown (A, C, E) preoperative and (B, D, F) postoperative at frontal, lateral, and anterior oblique views.

**Figure 5.**
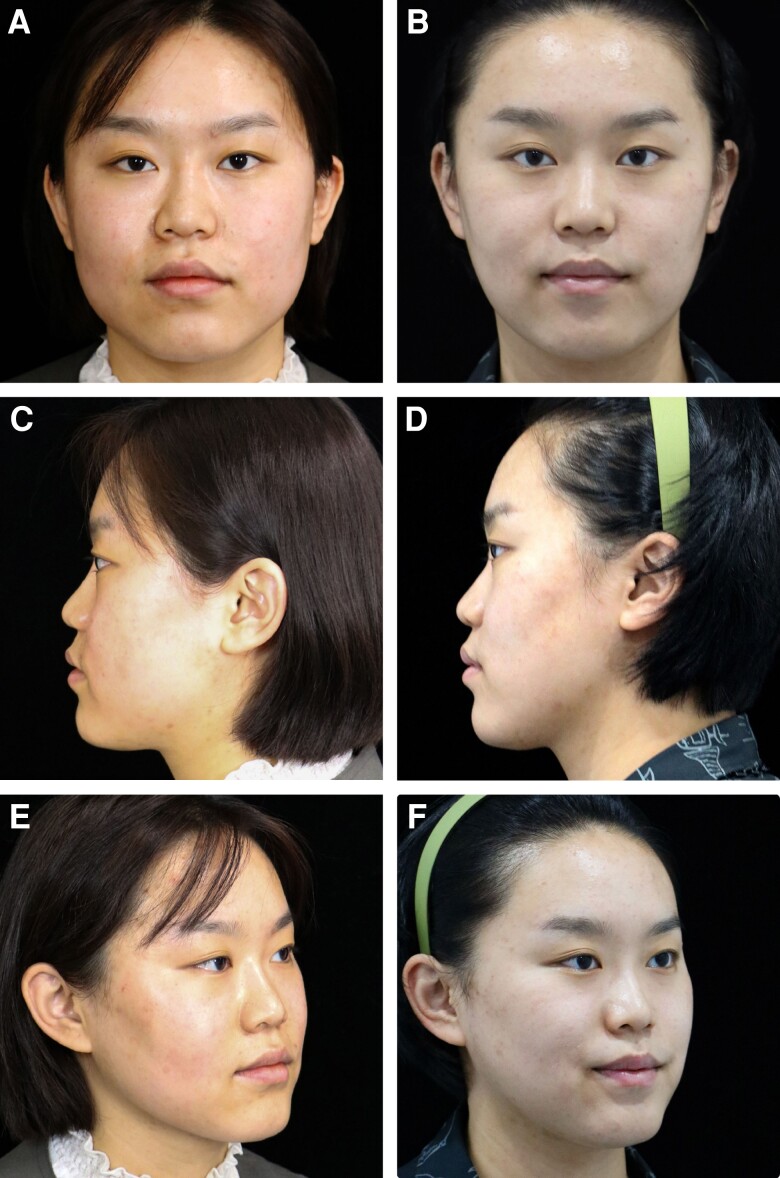
A 26-year-old female patient with lower face and neck heaviness and poorly defined jawline and mild skin ptosis shown (A, C, E) preoperative and (B, D, F) postoperative at frontal, lateral, and anterior oblique views.

In 60 Knize Grades II and III patients, the posttreatment incidences of Knize Grades I, I and II, II, and II and III were 8, 50, 1, and 1, respectively. In 4 Knize Grade III patients, the posttreatment incidences of Knize Grades I and II and II were 1 and 3, respectively. In 2 Knize Grade IV patients, the posttreatment incidences of Knize Grades II and II and III were 1 and 1, respectively (Table 3). After RFAL treatment, significant improvement in the cervicomental angle definition was observed, as shown in Figures 2‐5.

**Table 3.** Results of Knize Scale Grading of Cervicomental Angle Between Preoperative and Postoperative Photographs

Preoperative grade	Postoperative grade	No. of patients
II-III (60, 90.91%)	I	8
	I-II	50
	II	1
	II-III	1
III (4, 6.06%)	I-II	1
	II	3
IV (2, 3.03%)	II	1
	II-III	1

**Figure 6.**
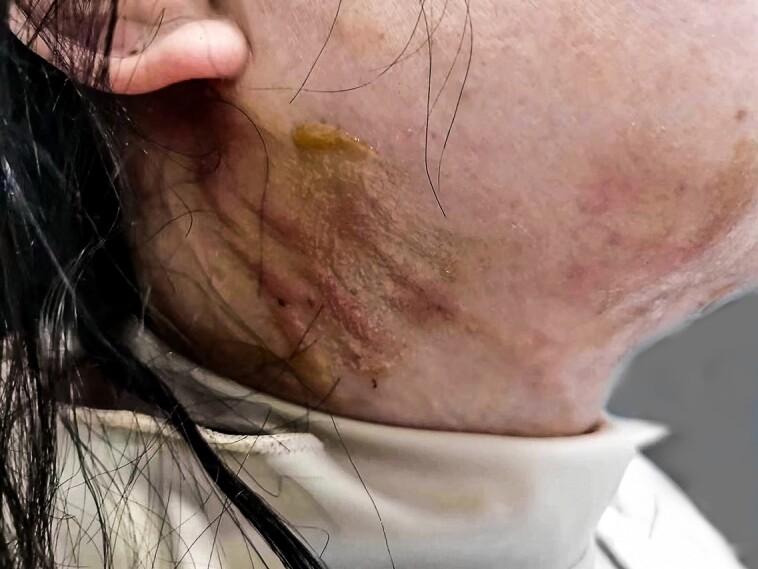
A 33-year-old female patient presenting with second-degree cervical skin thermal injury on postradiofrequency-assisted liposuction treatment Day 1.

**Figure 7.**
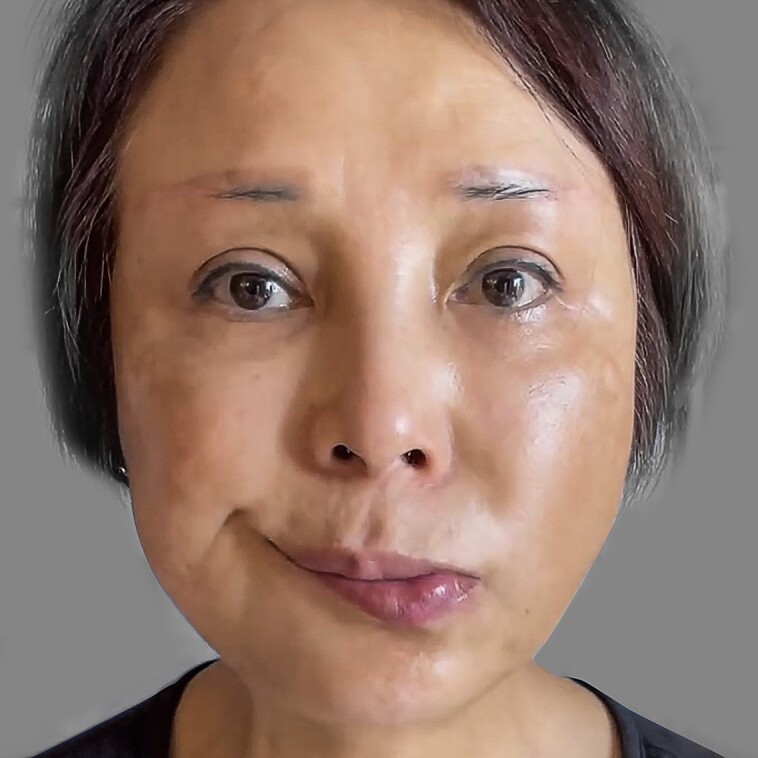
A 45-year-old female patient presenting with temporary unilateral neuropraxia of the marginal branch of the mandibular nerve on postradiofrequency-assisted liposuction treatment Day 3.

There were no major complications or adverse events requiring further medical or surgical intervention. None of the patients had hematoma, infection, permanent motor nerve damage, or seroma after RFAL treatment. In terms of minor complications, 5 (7.58%) patients presented with first- and second-degree burns; 3 (4.55%) with temporary unilateral neuropraxia, as shown in Figures 6 and 7; and 5 (7.58%) with nodules during post-RFAL recovery treatment. After treatment with RFAL, the mean GAIS score was 3.03, patient's satisfaction score was 3.41, and the subjective effectiveness rate was 93.94% (33 with an excellent rating and 29 with a good rating).

A 31-year-old female patient presented with a BMI of 19.5 had cervical skin laxity, a lack of lateral jaw angle projection, and submental fat accumulation leading to lower face heaviness. The cervicomental angle was blunted. Postoperative views 6 and 12 months after RFAL treatment demonstrated substantial improvement of the cervicofacial region, a slender lower face shape, and improvement in the geometric angle between the cylindrical neck and the straight-line inferior border of the jaw. FaceTite device: external probe temperatures setting, 38.0°C; internal probe temperatures setting 62.0°C, total energy delivered, 5.0 kJ; operative duration 80 min; total aspiration volume, 20.0 mL (Figure 2).

A 32-year-old female patient presented with a BMI of 20.5, an obtuse cervicomental angle, and a lack of lateral jaw angle projection with a poorly defined jawline. Postoperative photos obtained at 6 and 12 months demonstrated substantial improvement in the cervicomental angle definition and a more demarcated gonial angle and mandibular line. Bipolar RFAL was performed under tumescent local anesthesia. FaceTite device: external probe temperatures setting, 38.0°C; internal probe temperatures setting, 68.0°C; total energy delivered, 6.3 kJ; surgery duration 75 min; total aspiration volume, 20.0 mL (Figure 3).

Preoperative scan of a 47-year-old female patient with a BMI of 22 showing a poorly defined cervicomental angle. The neck appears heavy and short. After 6 months of RFAL treatment, the cervicomental angle is refined and the neck appears longer and thinner. Bipolar RFAL was performed under tumescent local anesthesia. FaceTite device: external probe temperatures setting, 38.0°C; internal probe temperatures setting, 68.0°C; total energy delivered, 7.0 kJ; surgery duration 55 min; total aspiration volume, 25.0 mL (Figure 4).

A 26-year-old female patient with a BMI of 25.3 kg/m^2^ presented with lower face and neck heaviness and poorly defined jawline. Six months after RFAL treatment of the lower face and neck, the face slimmed down, and the jawline became more demarcated. Bipolar RFAL was conducted under tumescent local anesthesia. The FaceTite device was used under the following settings: external probe temperature, 40.0°C; internal probe temperature, 66.0°C; total energy delivered, 6.6 kJ; surgical duration, 70 min; and total aspiration volume, 50.0 mL (Figure 5).

A 33-year-old female patient presented with superficial second-degree burn after RFAL treatment of the neck (Figure 6). A 42-year-old female patient had unilateral marginal branch of the mandibular nerve neuropraxia (Figure 7).

## DISCUSSION

We found that the use of bipolar RFAL for neck skin and soft-tissue tightening among Eastern Asian subjects achieved extraordinary results. From the postoperative results, it is apparent that the cervicomental angle definition became more and more visible over time.

Interestingly, although most subjects (90.91%) only had RFAL treatment on the cervical region (from the submandibular region down to the level of the thyroid cartilage and the anterior border of the SCM), not only did their cervicomental angle definition improve but their mandibular line and the gonial angle projection also progressively became more demarcated. Moreover, the loosened skin of their lower face was also tightened, making the face more oval shaped. The postoperative results were very appealing to East Asians’ aesthetic preferences. It is also worth mentioning that in the East Asian female population, several patients presented with microgenia or recessed chins.^15^ Further, the redundant submental fat and tissue increases with age and blunts the cervicomental angle definition. In patients with microgenia or recessed chins with redundant fat and soft tissue who want face and neck rejuvenation, submental liposuction combined with concurrent genioplasty is more effective. In this case series, the operators (SC.Y. and JG.W.) observed that the skin-tightening effect of RFAL was remarkable compared with that of traditional liposuction in patients with microgenia. This might be attributed to the extra contraction and retraction ability of RF energy to ablate soft tissue and tighten the overlying skin. Duncan^9^ showed that RF application combined with SAL was effective in achieving greater skin surface area reduction. In her study, the 12-month skin contraction rates were 34.5% for SAL combined with RFAL treatment and 8.3% for SAL alone. Therefore, RFAL treatment might be beneficial in patients with microgenia combined with skin and soft-tissue laxity who want face and neck tightening. The deployment of RFAL may become an alternative option in patients who refuse genioplasty or mandibular advancement surgery but want to improve lower face and cervical tissue laxity. However, more studies should be conducted to validate these data.

There are different opinions in the literature regarding the surgical steps involved in RFAL. In most studies, surgeons deployed RF applications prior to SAL.^10,16,17^ In contrast, Han et al proposed a 2-step method, which entailed performing SAL before RFAL.^11^ This method allowed the surgeon to establish the correct RF working channel, expose the fibroseptal network tissue by SAL, and apply more RF energy directly targeting the fibroseptal network tissue to achieve a more efficient skin-tightening effect. The authors agreed that Han's approach led to better postoperative results. Additionally, since the tumescent fluid has a cooling impact on the treatment area, by aspirating the infiltrated tumescent fluid, the underlying subcutaneous tissues would have a better thermal effect. Moreover, based on the 2-step method, the authors suggested evacuating in situ nonvital tissues after RFAL because a previous study on breast augmentation indicated that the necrotic fat caused chronic inflammation and progressive fibrosis response during the healing process.^18^ Furthermore, the nature of the skin of Eastern Asians tends to make it have a more vigorous fibroplastic response during the wound-healing process,^19^ which potentially contributes to postoperative nodules and induration. The extra evacuating maneuver will decrease the postoperative focal chronic inflammatory response, reduce the occurrence of postoperative nodules and induration, and accelerate the wound-healing process.

In this case series, all 5 thermal injuries were mild (first- and second-degree burns) and located around the skin entrance area. Hence, any medical intervention was not provided. Notably, all types of burns were observed in our first 5 cases when the operators were learning the movement (retrograde and anterograde strokes and stamping) of the bipolar probe. Subsequently, we found that thermal injury was caused by the tenting of the internal probe (Facetite handpiece) to the skin during anterograde strokes and repeated heating around the entrance area. Thermal injuries can be prevented by constantly moving the RFAL probes slowly horizontal to the skin, using the retrograde approach, and providing a few millimeter space between the internal RF probe and the subdermis. Mild thermal injuries were treated within 3 to 4 weeks without medical intervention.

In our study, 3 patients presented with transient marginal mandibular nerve neuropraxia during recovery from RFAL. The “wry mouth” symptom was reported immediately after the RFAL treatment and 2 of them achieved full recovery in 1 week, whereas the remaining patient recovered in 4 weeks without other adverse effects. This finding is consistent with previous reports that nerve recovery occurs after remyelination and that sensorimotor functions can usually be fully restored within days to weeks without sequelae.^20,21^ Neuropraxia was caused by temporary interruption in the conduction of nerve impulses and motor function, which was a consequence of trauma to the nerve fibers without nerve rupture. Given that the marginal nerve was dominant for lower lip depressor function, temporary unilateral neuropraxia of the marginal branch of the mandibular nerve resulted in paralysis of the ipsilateral depressor anguli oris muscle. When performing RFAL to the neck, it is not recommended to go above the mandible and medial to the marionette lines where the marginal mandibular nerve is typically found. However, the position of the marginal mandibular nerve with respect to the mandibular border varies, and it is often asymmetrical. To prevent nerve injuries, DiBernardo et al showed the importance of mapping the marginal mandibular nerve prior to performing RF skin-tightening procedures on the neck to prevent nerve trauma.^22^

Finally, further studies with a high number of participants and SAL control group should be performed. Further, an objective evaluation should be conducted using a more objective observation indicator in addition to the GAIS score. The main limitations of this study were related to its retrospective design and the absence of a control group with SAL treatment. The results could also be affected by the nonuniformity of treatment parameters. Moreover, the number of included patients was small, and analyses of patients’ age groups and skin type characteristics were not performed. However, despite these limitations, these data provided valuable insights about RFAL treatment applied to cervical contouring for Eastern Asians, and the findings of this study may serve as a basis for future studies. Further large-scale prospective studies should be performed to verify the findings of this study and optimize RFAL treatment parameters.

## CONCLUSIONS

We found that RFAL treatment, when performed with the recommended settings mentioned in this paper, is effective in neck contouring, especially in achieving geometric angles between the cylindrical neck and the straight-line inferior border of the jaw that East Asian patients widely seek. The RFAL treatment studied in this paper showed an apparent improvement in refining neck contouring among Eastern Asian subjects. Moreover, this single, 1-time minimally invasive cervical procedure under tumescent local anesthesia not only improves the cervical-mental angle definition but also causes the tissue-tightening effect, face slimming, and the mandibular line and the gonial angle projection to progressively become well demarcated. We believe that this application could achieve extraordinary skin and soft-tissue-tightening outcomes with a high safety profile if done correctly.
